# Relapse or reinfection after failing hepatitis C direct acting antiviral treatment: Unravelled by phylogenetic analysis

**DOI:** 10.1371/journal.pone.0201268

**Published:** 2018-07-25

**Authors:** Lize Cuypers, Ana Belén Pérez, Natalia Chueca, Teresa Aldamiz-Echevarría, Juan Carlos Alados, Ana María Martínez-Sapiña, Dolores Merino, Juan Antonio Pineda, Francisco Téllez, Nuria Espinosa, Javier Salméron, Antonio Rivero-Juarez, María Jesús Vivancos, Víctor Hontañón, Anne-Mieke Vandamme, Féderico García

**Affiliations:** 1 KU Leuven–University of Leuven, Department of Microbiology and Immunology, Rega Institute for Medical Research, Clinical and Epidemiological Virology, Leuven, Belgium; 2 Peter Medawar Building for Pathogen Research, Nuffield Department of Medicine, University of Oxford, Oxford, United Kingdom; 3 Clinical Microbiology Department, University Hospital San Cecilio Granada, Instituto de Investigación Ibs. Granada, Spain; 4 Infectious Diseases Unit, Hospital Gregorio Marañón, Madrid, Spain; 5 Clinical Microbiology, University Hospital Jerez, Cadiz, Spain; 6 Clinical Microbiology, Hospital Miguel Servet, Zaragoza, Spain; 7 Clinical Microbiology, Hospital Infanta Elena, Huelva, Spain; 8 Infectious Diseases Unit, University Hospital de Valme, Sevilla, Spain; 9 UGC Enfermedades Infecciosas y Microbiología, Hospital La Línea, AGS Campo de Gibraltar, Cadiz, Spain; 10 Clinical Microbiology, Hospital Virgen del Rocío, Sevilla, Spain; 11 Hepatology Unit, University Hospital San Cecilio Granada, Instituto de Investigación Ibs. CIBERehd, Granada, Spain; 12 Infectious Diseases Unit. Instituto Maimonides de Investigación Biomédica de Córdoba (IMIBIC). Hospital Universitario Reina Sofía de Córdoba. Universidad de Córdoba, Córdoba, Spain; 13 Clinical Microbiology, Hospital Ramón y Cajal, Madrid, Spain; 14 Clinical Microbiology, University Hospital La Paz, Madrid, Spain; 15 Center for Global Health and Tropical Medicine, Microbiology Unit, Institute for Hygiene and Tropical Medicine, University Nova de Lisboa, Lisbon, Portugal; University of Cincinnati College of Medicine, UNITED STATES

## Abstract

Despite high response rates associated to hepatitis C virus (HCV) treatment, no protective immunity is acquired, allowing for reinfection and continued infectiousness. Distinguishing between relapse and reinfection is crucial for patient counselling and to choose the most appropriate retreatment. Here, refined phylogenetic analysis using multiple genes served to assess genotype and reinfection for 53 patients for whom the virus was sampled before start of therapy and at time of sustained virological response evaluation at week 12. At baseline, genotypes were determined as HCV1a (41.5%), HCV1b (24.5%), HCV4 (18.9%) and HCV3a (15.1%), while six cases revealed to be discordantly assigned by phylogeny and commercial assays. Overall, 60.4% was co-infected with HIV. The large majority was classified as people who inject drugs (78.6%), often co-infected with HIV. Transmission was sexual in seven cases, of which five in HIV-positive men-who-have-sex-with-men. Overall, relapse was defined for 44 patients, while no conclusion was drawn for four patients. Five patients were reinfected with a different HCV strain, of which three with a different genotype, showing that phylogeny is needed not only to determine the genotype, but also to distinguish between relapse and intra-subtype reinfection. Of note, phylogenies are more reliable when longer fragments of the viral genome are being sequenced.

## Introduction

Despite occasional claims of integration events [1], the hepatitis C virus (HCV) is considered to have no latent forms and to rely on ongoing replication. Therefore, in contrast to HIV, patients infected with HCV can either spontaneously clear their infection or can be cured thanks to treatment with highly effective antiviral regimens which are nowadays based on molecules that directly target viral proteins, the so called direct-acting antivirals (DAAs). Viral cure rates have dramatically increased to 90–95% following the introduction of interferon-free (IFN-free) DAA combination therapies [2]. Although less crucial than during the IFN era, the HCV genotype (GT) still plays a substantial role in deciding the best treatment regimen of a patient [3], hand in hand with the respective stage of liver fibrosis or cirrhosis and the previous experience of the patient to IFN-based treatment regimens.

In spite of high treatment response rates, no protective immunity is built [4], so patients that are cured at the end of therapy can be infected with a new HCV strain, and might be still at risk to develop liver diseases [5], and to form a source of further transmission events. Patients reinfected with a strain determined to be of a different genotype or subtype than the previous strain they were infected with, can easily be identified using genotyping assays. However, when a reinfection with a similar strain of the same subtype occurs, phylogenetic analysis is required to distinguish a reinfection from a virological relapse [6–7]. While iatrogenic infected HCV patients are expected to clear their infection and to remain cured, viral eradication is not expected soon at global level, given the high number of individuals that is unaware of their infection status, the low treatment uptake and the continued *de novo* HCV incidence that in many countries exceeds the rate of curative treatment. Especially in specific populations such as people who inject drugs (PWID) and HIV-positive men who have sex with men (MSM), viral eradication is hampered by the assumed high reinfection and transmission rates after successful DAA treatment. However, wide ranges in the number of reinfection cases have been reported for these risk groups [8–9], depending on the patient population studied, the treatment era in which the study was planned and if reinfection followed viral clearance due to the immune system or due to a prescribed treatment regimen. In general, reinfection rates are reported to be lower for PWIDs, although depending on their active drug use [10–11], compared to HIV-positive MSM [10,12–13]. For PWIDs, generally rather low reinfection rates from 0 to 5 cases per 100 person-years were reported [13–14], although rates can go up to 10% [9]. In case of patients that are co-infected with HIV, of which the majority are MSM, rates easily increase to 15–25% [10,15]. Moreover, recently also a high prevalence of HCV infection has been reported in HIV-negative MSM that are enrolled for HIV pre-exposure prophylaxis [16]. Distinguishing between virologic relapse and infection with a new viral strain is highly important to determine the true treatment efficacy of the current DAA regimens, to define the most appropriate retreatment for these patients [17], and more importantly for appropriate patient counselling. Treatment scale-up, diagnostic testing approaches and strategies to prevent new infections as well as reinfections, need to occur concomitantly to accomplish worldwide viral eradication of HCV.

## Methods

### Inclusion of patients from the GEHEP-004 cohort

HCVREsp-GEHEP004 is a prospective multicentre cohort including 7189 HCV infected patients treated with IFN-free DAA regimens, attending 54 different Spanish centres, all part of the Group for the Study of Viral Hepatitis (GEHEP). So far, this cohort enrolled around 450 patients that were unsuccessfully treated with DAAs (update from [18]). From this last group, in 53 patients, samples at two time points were referred for resistance testing, and all were included in this analysis. For all other patients, only one of these two time points was sampled. The patients included in this study were infected with various HCV GTs and were treated between 2014 and 2016 with the standard of care DAA regimens at that time, in Spain. In total, 37.7% of all patients (20/53) failed previous therapy based on sofosbuvir + ledipasvir, with or without ribavirin, followed by a treatment consisting of three DAA classes (3D: paritaprevir + ombitasvir + dasabuvir + ribavirin) for 20.8% of the patients (11/53). Next in line was the regimen based on sofosbuvir + daclatasvir + ribavirin. A lower number of patients had initiated therapy with sofosbuvir + simeprevir + ribavirin, simeprevir + daclatasvir + ribavirin, sofosbuvir + ribavirin or the 2D combination (without dasabuvir) + ribavirin (S1 Table). The relatively high therapy failure rate can be attributed to the use of inferior regimens during the early days of DAA therapy in 2014, and due to the incorrect determination of HCV genotypes using commercial assays, resulting in the choice of a potential suboptimal regimen.

### Genetic sequencing

HCV was sampled at two time points for each patient, before start of treatment (at baseline) and at time of sustained virological response evaluation at week 12 (further on referred to as at SVR12 evaluation– 12 weeks after end of treatment). All patients had an undetectable viral load at the end of treatment, hence why the detection of virus 12 weeks after end of treatment either indicates the occurrence of reinfection or relapse, the latter considered as treatment failure [19]. In the context of drug resistance testing, genetic sequencing was performed for three different genetic regions in the HCV genome, more particularly NS3, NS5A and NS5B, routinely being sequenced using *in house* developed assays, respectively amplifying 513, 258 and 312 nucleotides. Depending on the composition of the administered DAA regimen, all three, or only one or two genes, were sequenced. For the purpose of this study, retrospective Sanger sequencing was performed to recover the genetic sequence of at least two regions for as many patients as possible. For the cases here distinguished as a reinfection, next-generation sequencing (NGS) was performed on the baseline and follow-up sample of the respective patient, starting from the same NS5B PCR product as obtained for Sanger sequencing, to rule out the occurrence of a mixed infection at the start of treatment and/or at time of SVR12 evaluation. Until the end of 2016, the 454 GS Junior pyrosequencing methodology (Branford, Connecticut, USA) was used to perform NGS, however afterwards the Illumina platform (San Diego, CA, USA) was applied, based on a modified protocol described in [20]. The HCV genotype of all individual reads was determined to rule out the occurrence of a mixed infection. All sequences generated within this study, have been submitted to Genbank (accession numbers MG983221-MG983474). The Ethics Committee of the San Cecilio Hospital, Granada, approved the study, sequencing experiments were performed in accordance to good laboratory practices, and no informed consent was required as patient information was anonymized prior to analysis.

### Determination of the HCV genotype and between-subtype or genotype recombination

Genotypes were determined using the commercial assay Versant HCV Genotype 2.0 assay (LiPA) for the majority of the baseline samples (86.8% or 46/53), while the genotype of 9.4% (5/53) and 3.8% (2/53) of the baseline samples was defined by the Abbott Real Time HCV Genotype II assay and Trugene HCV Genotyping Kit, respectively. The HCV genotype and subtype of the samples was also determined by manual phylogenetic analysis and the use of subtyping tools COMET and Oxford [21–22]. In case of discordance between the different tools, a bootscan analysis with Simplot was performed to assess potential breakpoints for recombination [23]. Resistance-associated substitutions (RASs) were evaluated for the NS3, NS5A and NS5B sequences at time of SVR12 evaluation, based on variants reported in literature [24].

### Dataset construction and assessment of within-subtype recombination

The ten most similar sequences to each of the 53 Spanish taxa, were retrieved by the use of the standalone BLAST tool [25]. The resulting datasets were constituted based on the fragment that was sequenced, more specifically resulting in seven datasets, either consisting of sequences coding for one fragment (NS3, NS5A, NS5B) or concatenated in case more than one region was sequenced (NS3-NS5A-NS5B, NS3-NS5A, NS3-NS5B and NS5A-NS5B). The latter strategy increased the genomic fragment length, and therefore the phylogenetic signal. Sequences were aligned per dataset and manually edited to assure high quality, using Seaview and MEGA 7.0 [26–27]. An extensive recombination analysis was done using RDP4 and TreePuzzle [28–29]. The likelihood-mapping algorithm in TreePuzzle was used to assess the percentage of conflicting phylogenetic signal, represented by the dots at the sides of the triangle. Potential recombination events indicated by RDP4 were only confirmed if two parental sequences could be identified, and if breakpoints were significant. Since this is depending on the strains included in the analysis, absence of evidence of within-subtype recombination might not be equal to evidence of absence of within-subtype recombination.

### In-depth phylogenetic analysis

The resulting codon-correct alignments were inferred by neighbour-joining (NJ) and maximum-likelihood (ML) approaches, applying the GTR gamma model to allow for among-site variation, and evaluating tree robustness with 1000 bootstrap replicates. For the NJ approach, trees were inferred as implemented in MEGA 7.0, while for the (approximate) ML algorithm, trees were constructed using FastTree and RAxML [30–31]. Additionally, positions known to be impacted by RASs [24], were removed from the alignment, to evaluate them as potential source of confounding in the inference of tree topologies.

When comparing strains before and after treatment in the same patient, evidence of reinfection was defined as a difference in HCV genotype or subtype, or as a significantly different clustering located in different clades in the phylogenetic tree. Evidence of virological relapse was defined as significant clustering in the same clade, while no conclusion was drawn when clades were supported with a bootstrap <70%. Despite this predefined threshold, the lowest bootstrap value to define a relapse was found to be 83%.

## Results

### Predominance of HCV1a infection, PWIDs and HIV co-infection

At baseline, 22 out of 53 patients (41.5% or 22/53) were phylogenetically determined to be infected with HCV1a, followed by HCV1b (24.4% or 13/53), HCV4 (18.9% or 10/53: 7.5% 4a – 11.3% 4d), and HCV3a (15.1% or 8/53). A large share of patients was co-infected with HIV (60.4% or 32/53). The transmission route of infection was known for 79% of patients (42/53), with all patients lacking information for risk of transmission being mono-infected with HCV. The large majority of patients with known route of transmission were reported to be (former) PWID (78.6% or 33/42), of whom 81.8% (27/33) was found to be co-infected with HIV and more than half of them with HCV1a (51.5% or 17/33). HCV transmission through sexual contact was inferred for seven patients, of which five were reported as MSM and two as heterosexual, all of them concomitantly infected with HIV. One patient was infected through blood transfusion during childhood and another one during haemodialysis. For none of the patients, a dual route of transmission was reported in the clinical database.

### HCV genotype misclassifications

HCV genotype and subtype assignment by phylogenetic analysis and the use of well-known subtyping tools was in agreement with the assignment by commercial assays for 66.0% of all baseline samples (35/53). The majority of misclassifications by commercial assays was due to assignments solely on genotype level, lacking information on subtype level. However, also six baseline samples were wrongly classified on HCV genotype and subtype level (S1 Table). The sequence at baseline for one patient of the cohort was concordantly identified to be HCV1a by all genotype determination methods, while the strain at time of SVR12 evaluation was assigned as HCV1a or HCV1b, dependent on the genetic region analysed and the determination method. In-depth analysis of the concatenated alignment covering regions NS5A and NS5B, could offer a definite classification of this patient’s sequence at time of SVR12 evaluation as HCV1a, excluding the occurrence of an inter-subtype recombination event. Moreover, in none of the 53 patients in the study, evidence of a recombination event was identified using Simplot or RDP4. The ability to detect recombination was evaluated using TreePuzzle, showing only a small percentage (0.3–0.5%) of phylogenies characterized by conflicting signal. Evaluating RASs, either naturally occurring or substituted under drug selective pressure, showed that patients in this study mainly harboured variants in the NS3 and NS5A genes, while only for three patients RASs were detected in NS5B (282T/R). Two of the latter patients also harboured NS5A RASs, while in total 10 patients harboured dual variants in NS3 and NS5A. Single NS3 and NS5A variants were detected in 8 and 14 patients, respectively. Patients defined to have experienced a reinfection were analysed separately, showing that four out of five patients acquired NS5A RASs over the course of treatment, while for one of them already a natural variant was present at baseline. Details on the specific variants can be found in S2 Table.

### Phylogenetic analysis of concatenated alignments

Because of low phylogenetic signal when only one genetic region was used, additional genes were sequenced retrospectively. As a result, more than one genetic region was available for both time points sampled for all patients, except for two patients for which amplification was unsuccessful for regions NS3 and NS5B, respectively (S1 Table: patients 30 and 49). Therefore, while the tree suggested the occurrence of a relapse, this evidence was not conclusive since bootstrap values were <70%. However, for one of the two patients, reinfection is not highly likely since this patient declared to be infected by blood transfusion during childhood and to show no persistent risk behaviour to acquire a new HCV infection.

**Fig 1:**
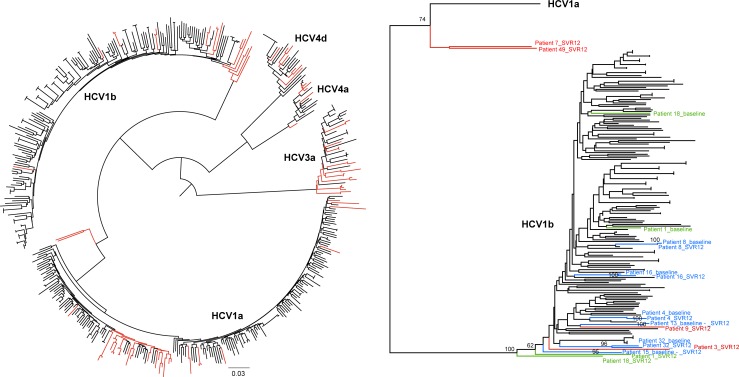
Maximum-likelihood tree of the concatenated alignment covering genetic regions NS5A and NS5B. Both the entire phylogenetic tree (left) and the HCV1b clade in detail (right) are visualized. Patient 18, coloured in green, is a clear example of reinfection with a different HCV strain, since although both viruses at baseline and at time of SVR12 evaluation are classified as HCV1b, they cluster in a different clade in the tree, with a bootstrap support of 100% for the segregation of the different clades. For patient 4, coloured in blue, both strains cluster together with a high bootstrap support (100%), suggesting that this patient experienced a virological relapse. Bootstrap replicates are only visualized for patients who experienced a relapse, all indicated in blue. Patients for which only a baseline or SVR12 evaluation sequence is included in the NS5A-NS5B alignment, are coloured in red. The sequence at time of SVR12 evaluation and baseline for patients 7 and 49 respectively, cluster outside the large HCV1b clade, which might be due to a potential event of recombination. However, detailed analyses with TreePuzzle and RDP4 for all 53 patients, could not support a recombination event. The in-depth Simplot analysis for patients 7 and 49 showed that both strains were classified as HCV1a. Despite the absence of evidence of recombination, we did not draw any conclusions concerning the occurrence of reinfection or relapse for patient 7. The bar at the bottom represents the number of nucleotide substitutions per site.

**Fig 2:**
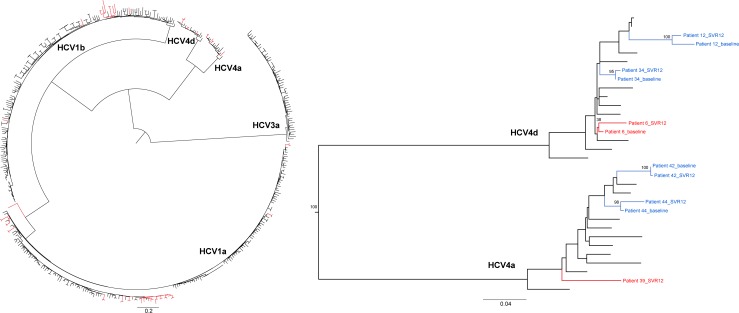
Maximum-likelihood tree of the concatenated alignment covering genetic regions NS3 and NS5A. Both the entire phylogenetic tree and the HCV4 (4a and 4d) clade in detail are visualized. Clusters of patients coloured in blue, are suggested to have experienced a virological relapse, supported by bootstrap values >70%. However, for patient 6, indicated in red, only a bootstrap value of 38 was obtained, resulting in lack of evidence to distinguish between a relapse and reinfection. For patient 39, the fragment NS3-NS5A was only sequenced for the sample at time of SVR12 evaluation. The bar at the bottom represents the number of nucleotide substitutions per site.

For all other patients, concatenated alignments were constructed. Phylogenetic inference using the four concatenated alignments (NS3 –NS5A –NS5B, NS3 –NS5A, NS3 –NS5B and NS5A –NS5B) showed that 44 out of 51 patients had experienced a virological relapse, while five (9.4%) were classified as reinfected with a different HCV strain (S1 Table). For three of these five patients (S1 Table: patients 5, 14 and 36), the sequences at baseline and at time of SVR12 evaluation differed in HCV genotype and/or subtype. NGS experiments ruled out the occurrence of a mixed infection at baseline and at time of SVR12 evaluation for all three patients. Two patients (S1 Table: patients 1 and 18) were reinfected with the same HCV subtype, phylogenetically clustering in a different clade for the two sampled time points (Fig 1). Of the five patients that were defined to be reinfected, three were classified as PWID, one identified himself as MSM and for one the potential route of transmission was unknown. Three out of these five patients were co-infected with HIV. For two additional patients, no conclusions could be drawn, either due to close clustering of both strains, however supported by bootstrap replicates lower than 70%, or due to an inconclusive clustering between the clades HCV1a and HCV1b (S1 Table: patients 6 and 7, Figs 1 and 2). Conclusions drawn from the phylogenetic analyses were not influenced by the removal of drug resistance-associated positions in the concatenated alignments.

## Discussion

Reinfection rates in real-life settings may be underestimated, especially when the patient is reinfected with the same subtype. Here we report how detailed phylogenetic analysis is needed to discriminate intra-subtype reinfection from relapse. This study demonstrates the importance of genetic sequencing, not only to define the most appropriate (re)treatment, but also to perform phylogeny needed to determine the correct HCV genotype and subtype that a patient is infected with and to distinguish between relapse and reinfection.

Concatenated genes were used to distinct between relapse and reinfection, however for two patients no conclusion could be drawn due to a bootstrap support <70% of the respective clade, or the inconsistent clustering between different HCV subtypes, respectively. Additionally, for two patients not more than one genetic region was successfully sequenced. For the other patients, the majority had a virological relapse (44/49). These patients are eligible for retreatment. Until recently, it was advised to choose the DAA regimen based on the presence of natural occurring or emergent RASs [2–3,32]. Since the approval of the new combinations consisting of sofosbuvir, velpatasvir and voxilaprevir, or glecaprevir and pibrentasvir, AASLD and EASL guidelines no longer routinely recommend RAS testing at time of failure. Moreover, in the era of true pan-genotypic combinations, the interest of determining the HCV genotype at baseline is diminishing as the prescribed regimen no longer depends on it. We hypothesize that in developed countries where first-line regimens will consist of the newest therapy combinations, baseline genotyping will be soon seen as obsolete. However, in the light of these changes, phylogenetic analysis used to determine viral failures as relapse or reinfection, will gain largely in importance, as even reinfections with a different genotype as the strain of the first infection, would be missed.

Almost 10% of patients (5/53) were found to be reinfected with a different HCV strain, two of which (2/5 or 40% of reinfections, while 2/53 or 3.8% of all patients) would have been missed in absence of phylogenetic analysis since the sequences at baseline and time of SVR12 evaluation were of the same HCV subtype. Phylogenetic analysis confirmed reinfection in three patients with discordant genotype or subtype. Three out of these five reinfected patients reported to be infected by intravenous drug use, while one was an MSM, and for the fifth patient no route of transmission was known. Additionally, three of them were known to be co-infected with HIV. It has been reported that equipment used during, before, or after sexual activity [33], high-risk sexual behaviour in general [34–35], as well as the use of drugs [36–37], could be associated to a higher risk of HCV transmission, showing a potential role of multifactorial risk behaviour instead of transmission through one dominant route.

The main limitation of this study was that it was not specifically designed to evaluate reinfection rates; as the aim of the GEHEP cohort is to evaluate the emergence of RASs at failure. Moreover, the selection of 53 patients out of a cohort of 450 may have introduced a bias with respect to a population-based analysis, however these were the only patients for which samples at two time points were available. Since this was a random set of 53 patients, the general characteristics did not differ from the other 397 patients. Complete information regarding the risk of transmission is unavailable for the remaining 397 patients, not allowing us to evaluate the extent of this potential bias. Additionally, a large share of the study population (>60%) was observed to be co-infected with HIV, which is much higher than reported for the overall HCV infected population in Spain [38]. Nevertheless, we believe these limitations did not heavily impact our main conclusion, supporting the added value of phylogeny to rule out intra-subtype reinfection.

Various genes of the HCV genome have been subjected to phylogenetic analysis, with the envelope genes E1 and E2 dominating the study landscape [39–41], more particularly hypervariable region-1 as this region is characterized by a high evolutionary rate [42]. However, in the context of treatment follow-up, these genes are not routinely sequenced, in contrast to proteins NS3, NS5A and NS5B. Despite the benefit of a higher phylogenetic signal, the added value of using viral genetic sequences obtained in clinical practice outranges the use of a more phylogenetically applicable gene such as E2, at least in the context of our study. Moreover, recent studies tend to use more often the NS3, NS5A and/or NS5B genes for phylogenetic purposes, although associated to a lower degree of genetic variability [17,43].

Phylogeny of the separate genetic regions often resulted in inconsistencies between the different examined genes, due to unresolved phylogenies. Especially when using the single fragment NS5A, phylogenies consisted of branches supported with low bootstrap values, since these respective sequences only covered 76 amino acid positions. It is therefore worrying that the rise in large-scale genetic sequencing in the context of clinical follow-up and drug resistance testing, has been and still is targeting only a limited number of amino acid sites in the HCV genome. Such data cannot be used to investigate reinfection, instead, longer regions such as full-length genomes are needed to obtain more robust phylogenies, as most often this approach highly improves the phylogenetic signal [17,40–41]. When concatenating multiple genes, combining regions characterized by a different evolutionary rate has been proposed to increase the capacity to identify transmission chains [44], while others underline that there is no ultimate gene to perform evolutionary analyses of genetically diverse viruses [45]. Fortunately, since HCV rarely recombines [46], at least studied on inter-genotype or inter-subtype level, thorough analyses using multiple genes should still have a genome-wide representativeness. In our dataset, we did not observe evidence of recombination, neither inter- nor intra-subtype or–genotype.

Less than 70% of HCV genotype assignments by commercial assays appeared to be consistent with well-known subtyping tools and phylogenetic analysis. Although the majority of misclassifications by commercial assays was due to an incomplete assignment on subtype level, still six baseline samples (11%) were wrongly classified on HCV genotype and/or subtype level, which may have potentially resulted in the choice of a less effective DAA therapy for these patients, as they were treated between 2014 and 2016. Commercial assays are known to be unreliable in case of mixed infections or recombination events since they only target a limited region of the HCV genome. Inconsistencies between commercial assays and genetic sequencing followed by phylogeny as HCV genotyping tool have also been reported in absence of recombination, ranging around 10% of misclassifications [47–49]. This study confirms the added value of genetic sequencing next to (or potentially instead of) the use of commercial assays in the assessment of HCV genotypes, as well as to acquire additional information on the presence of RASs which have proven to reduce susceptibility to certain antiviral drugs [50]. Notably, NGS was performed on the samples of both time points from patients identified as being reinfected, to rule out the presence of a mixed infection, as studies in the pre-DAA era have shown that the emergence of new viral strains following therapy failure is often associated to the emerging dominance of pre-existing minority variants rather than an actual reinfection [51].

The observation that a large part of the infections was assigned to be GT1 (66%) is overall in agreement with a large Spanish study on HCV genotype prevalence and distribution [38]. However, in our study a higher proportion of cases was infected with HCV1a and HCV4 compared to the cohort of [38], potentially due to the difference in population characteristics, since our study has a larger share of HIV/HCV co-infected patients (60.4% versus 19.1%). For these patients, both intravenous drug use and sexual transmission have been defined as the major routes of transmission, for which a higher proportion of HCV1a and HCV4 infections was reported compared to the overall population [38]. Especially HCV1a is more commonly observed among HCV infected PWIDs [52]. The majority of patients in this study were reported to be infected through this transmission route, in agreement with a rising HCV epidemic dominated by PWIDs in Spain, as reported previously [53]. All patients for which the route of transmission was unknown, were identified to be only infected with HCV and not with HIV, stressing the added value of capturing information concerning the potential route of transmission, as done in HIV clinical care.

In this cohort of unsuccessfully treated patients, the majority experienced a true DAA failure. Since about half of those reinfected, showed the same subtype as at baseline, phylogenetic analysis is needed, not only to determine the correct HCV genotype, but also to distinguish between relapse and reinfection. Given that 11% of genotypes were misclassified using commercial assays, and that 4% had a same-subtype reinfection only detectable by phylogeny, the potential of misclassifying a reinfection as a failure could be as high as 15%.

## Supporting information

S1 Graphical Abstract Graphical overview of the study cohort, methodology and results. (SVG)

S1 Table Overview of the assignment of virologic relapse or reinfection for the Spanish cohort. For each of the 53 patients in the cohort, the genetic region(s) that was or were sequenced for both time points is (are) listed, as well as the HCV genotype and subtype determined for the patient. In case of a misclassification of the HCV genotype by a commercial assay (excluding the ones due to commercial assays not classifying down to subtype level), cells are marked in grey. Phylogenetic analysis showed either evidence for virologic relapse or reinfection, although for four patients no conclusion could be drawn due to lack of phylogenetic signal or bootstrap values <70% or inconsistent clustering, also in this case the cell is colored grey.
(DOCX)

S2 Table Overview of the frequency and distribution of resistance-associated substitutions. For all 53 patients, resistance-associated substitutions (RASs) were listed at baseline and at time of SVR12 evaluation, in parallel to HCV genotype information, HIV co-infection status and classification of reinfection or relapse. For the latter, the symbol ‘?’ indicates that phylogenetic analysis could not resolve whether a relapse or reinfection occurred. In case no genetic sequencing was performed for a particular gene, this was indicated by the symbol ‘-‘ in the cell, while ‘None’ states that no RASs were detected.
(DOCX)
