# Supplementary material for: Relapse or reinfection after failing hepatitis C direct acting antiviral treatment: Unravelled by phylogenetic analysis
Source: PLoS One. 2018 Jul 25;13(7):e0201268. doi: 10.1371/journal.pone.0201268 (PMC6059487; doi:10.1371/journal.pone.0201268)
Supplement: S1 Table — For each of the 53 patients in the cohort, the genetic region(s) that was or were sequenced for both time points is (are) listed, as well as the HCV genotype and subtype determined for the patient. In case of a misclassification of the HCV genotype by a commercial assay (excluding the ones due to commercial assays not classifying down to subtype level), cells are marked in grey. Phylogenetic analysis showed either evidence for virologic relapse or reinfection, although for four patients no conclusion could be drawn due to lack of phylogenetic signal or bootstrap values <70% or inconsistent clustering, also in this case the cell is colored grey. (DOCX) [file pone.0201268.s002.docx]

**Relapse or reinfection after failing hepatitis C direct acting antiviral treatment: unravelled by phylogenetic analysis.**

Lize Cuypers^1,2,¶,*^, Ana Belén Pérez^3,¶^, Natalia Chueca^3^, Teresa Aldamiz-Echevarría^4^, Juan Carlos Alados^5^, Ana María Martínez-Sapiña^6^, Dolores Merino^7^, Juan Antonio Pineda^8^, Francisco Téllez^9^, Nuria Espinosa^10^, Javier Salméron^11^, Antonio Rivero-Juarez^12^, María Jesús Vivancos^13^, Víctor Hontañón^14^, Anne-Mieke Vandamme^1,15^, Féderico Garcia^3^

**S1 Table: Overview of the assignment of virologic relapse or reinfection for the Spanish cohort.** For each of the 53 patients in the cohort, the genetic region(s) that was or were sequenced for both time points is (are) listed, as well as the HCV genotype and subtype determined for the patient. In case of a misclassification of the HCV genotype by a commercial assay (excluding the ones due to commercial assays not classifying down to subtype level), cells are marked in grey. Phylogenetic analysis showed either evidence for virologic relapse or reinfection, although for four patients no conclusion could be drawn due to lack of phylogenetic signal or bootstrap values <70% or inconsistent clustering, also in this case the cell is colored grey.

| **Patient ID** | **Sampling time** | **Genetic region** | **HCV genotype** | **Conclusion phylogeny** |
| --- | --- | --- | --- | --- |
| Patient 1, 13 and 32 | Baseline | NS5A – NS5B | HCV1b | Reinfection |
|  | SVR12 evaluation | All three | HCV1b |  |
| Patient 2, 10, 11 and 53 | Baseline | NS5A – NS5B | HCV1a | Relapse |
|  | SVR12 evaluation | NS5A – NS5B | HCV1a |  |
| Patient 3 and 9 | Baseline | NS3 – NS5B | HCV1b | Relapse |
|  | SVR12 evaluation | All three | HCV1b |  |
| Patient 4 | Baseline | All three | HCV1b | Relapse |
|  | SVR12 evaluation | NS5A – NS5B | HCV1b |  |
| Patient 5 | Baseline | NS5A | HCV4d | Reinfection |
|  | SVR12 evaluation | NS5A – NS5B | HCV3a |  |
| Patient 6 | Baseline | NS3 – NS5A | HCV4d | Relapse or reinfection with a similar strain |
|  | SVR12 evaluation | All three | HCV4d |  |
| Patient 7 | Baseline | NS5A – NS5B | HCV1a | Probably reinfection |
|  | SVR12 evaluation | All three | HCV1a |  |
| Patient 8 | Baseline | NS5A – NS5B | HCV1b | Relapse |
|  | SVR12 evaluation | NS5A – NS5B | HCV1b |  |
| Patient 12 | Baseline | All three | HCV4d | Relapse |
|  | SVR12 evaluation | All three | HCV4d |  |
| Patient 14 | Baseline | NS5A – NS5B | HCV4d | Reinfection |
|  | SVR12 evaluation | All three | HCV1a |  |
| Patient 15 | Baseline | NS5A – NS5B | HCV1b | Relapse |
|  | SVR12 evaluation | NS5A – NS5B | HCV1b |  |
| Patient 16 | Baseline | All three | HCV1b | Relapse |
|  | SVR12 evaluation | All three | HCV1b |  |
| Patient 17, 20, 22, 23, 25, 26, 33, 38, 41 and 48 | Baseline | NS3 – NS5A | HCV1a | Relapse |
|  | SVR12 evaluation | All three | HCV1a |  |
| Patient 18 | Baseline | NS5A – NS5B | HCV1b | Reinfection |
|  | SVR12 evaluation | NS5A – NS5B | HCV1b |  |
| Patient 19, 24, 35, 45 and 47 | Baseline | NS5A – NS5B | HCV3a | Relapse |
|  | SVR12 evaluation | NS5A – NS5B | HCV3a |  |
| Patient 21 | Baseline | NS3 – NS5B | HCV4d | Relapse |
|  | SVR12 evaluation | NS3 – NS5B | HCV4d |  |
| Patient 27 | Baseline | NS5A – NS5B | HCV1a | Relapse |
|  | SVR12 evaluation | All three | HCV1a |  |
| Patient 28 | Baseline | NS5A – NS5B | HCV4a | Relapse |
|  | SVR12 evaluation | NS5A – NS5B | HCV4a |  |
| Patient 29 and 40 | Baseline | NS5A – NS5B | HCV3a | Relapse |
|  | SVR12 evaluation | NS5A – NS5B | HCV3a |  |
| Patient 30 | Baseline | NS3 – NS5A | HCV1b | Probably relapse |
|  | SVR12 evaluation | NS5A | HCV1b |  |
| Patient 31 | Baseline | NS3 – NS5A | HCV1b | Relapse |
|  | SVR12 evaluation | NS3 – NS5A | HCV1b |  |
| Patient 34 | Baseline | NS3 – NS5A | HCV4d | Relapse |
|  | SVR12 evaluation | All three | HCV4d |  |
| Patient 36 | Baseline | All three | HCV1a | Reinfection |
|  | SVR12 evaluation | NS5A – NS5B | HCV3a |  |
| Patient 37 | Baseline | NS5A – NS5B | HCV3a | Relapse |
|  | SVR12 evaluation | NS5A – NS5B | HCV3a |  |
| Patient 39 | Baseline | NS5A – NS5B | HCV4a | Relapse |
|  | SVR12 evaluation | All three | HCV4a |  |
| Patient 42 | Baseline | All three | HCV4a | Relapse |
|  | SVR12 evaluation | NS3 – NS5A | HCV4a |  |
| Patient 43, 50, 51 and 52 | Baseline | All three | HCV1a | Relapse |
|  | SVR12 evaluation | All three | HCV1a |  |
| Patient 44 | Baseline | All three | HCV4a | Relapse |
|  | SVR12 evaluation | All three | HCV4a |  |
| Patient 46 | Baseline | NS5A – NS5B | HCV1a | Relapse |
|  | SVR12 evaluation | NS5A – NS5B | HCV1a |  |
| Patient 49 | Baseline | NS5A – NS5B | HCV1b | Probably relapse |
|  | SVR12 evaluation | NS5A | HCV1b |  |
