# Supplementary material for: Relapse or reinfection after failing hepatitis C direct acting antiviral treatment: Unravelled by phylogenetic analysis
Source: PLoS One. 2018 Jul 25;13(7):e0201268. doi: 10.1371/journal.pone.0201268 (PMC6059487; doi:10.1371/journal.pone.0201268)
Supplement: S2 Table — For all 53 patients, resistance-associated substitutions (RASs) were listed at baseline and at time of SVR12 evaluation, in parallel to HCV genotype information, HIV co-infection status and classification of reinfection or relapse. For the latter, the symbol ‘?’ indicates that phylogenetic analysis could not resolve whether a relapse or reinfection occurred. In case no genetic sequencing was performed for a particular gene, this was indicated by the symbol ‘-‘ in the cell, while ‘None’ states that no RASs were detected. (DOCX) [file pone.0201268.s003.docx]

**Relapse or reinfection after failing hepatitis C direct acting antiviral treatment: unravelled by phylogenetic analysis.**

Lize Cuypers^1,2,¶,*^, Ana Belén Pérez^3,¶^, Natalia Chueca^3^, Teresa Aldamiz-Echevarría^4^, Juan Carlos Alados^5^, Ana María Martínez-Sapiña^6^, Dolores Merino^7^, Juan Antonio Pineda^8^, Francisco Téllez^9^, Nuria Espinosa^10^, Javier Salméron^11^, Antonio Rivero-Juarez^12^, María Jesús Vivancos^13^, Víctor Hontañón^14^, Anne-Mieke Vandamme^1,15^, Féderico Garcia^3^

**S2 Table: Overview of the frequency and distribution of resistance-associated substitutions.** For all 53 patients, resistance-associated substitutions (RASs) were listed at baseline and at time of SVR12 evaluation, in parallel to HCV genotype information, HIV co-infection status and classification of reinfection or relapse. For the latter, the symbol ‘?’ indicates that phylogenetic analysis could not resolve whether a relapse or reinfection occurred. In case no genetic sequencing was performed for a particular gene, this was indicated by the symbol ‘-‘ in the cell, while ‘None’ states that no RASs were detected.

| **Patient ID** | **Sampling** | **HCV GT** | **HIV** |  | **NS3 RAS** | **NS5A RAS** | **NS5B RAS** |
| --- | --- | --- | --- | --- | --- | --- | --- |
| Patient 1 | Baseline | HCV1b | No | Reinfection | - | 93H | None |
|  | SVR12 evaluation |  |  |  | 56F | 31V, 93H | None |
| Patient 2 | Baseline | HCV1a | Yes | Relapse | - | None | None |
|  | SVR12 evaluation |  |  |  | - | 31M | 282R |
| Patient 3 | Baseline | HCV1b | No | Relapse | 56F | - | None |
|  | SVR12 evaluation |  |  |  | 56F | None | None |
| Patient 4 | Baseline | HCV1b | Yes | Relapse | None | 31M | None |
|  | SVR12 evaluation |  |  |  | - | None | None |
| Patient 5 | Baseline | HCV4d | Yes | Reinfection | - | None | - |
|  | SVR12 evaluation | HCV3a |  |  | - | 93H | None |
| Patient 6 | Baseline | HCV4d | Yes | ? | None | 93H | - |
|  | SVR12 evaluation |  |  |  | None | 30C, 93H | 282T |
| Patient 7 | Baseline | HCV1a | Yes | ? | - | None | None |
|  | SVR12 evaluation |  |  |  | 168A/V | None | None |
| Patient 8 | Baseline | HCV1b | Yes | Relapse | - | 31M | None |
|  | SVR12 evaluation |  |  |  | - | 28T, 31M | None |
| Patient 9 | Baseline | HCV1b | No | Relapse | 56F | - | None |
|  | SVR12 evaluation |  |  |  | 56F, 168V | 31M | None |
| Patient 10 | Baseline | HCV1a | No | Relapse | - | None | None |
|  | SVR12 evaluation |  |  |  | - | 30H | None |
| Patient 11 | Baseline | HCV1a | Yes | Relapse | - | None | None |
|  | SVR12 evaluation |  |  |  | - | 30R | None |
| Patient 12 | Baseline | HCV4d | Yes | Relapse | None | None | None |
|  | SVR12 evaluation |  |  |  | None | None | None |
| Patient 13 | Baseline | HCV1b | ? | Relapse | - | 31M | None |
|  | SVR12 evaluation |  |  |  | 56F, 80K, 117H, 122T, 170I | None | None |
| Patient 14 | Baseline | HCV4d | Yes | Reinfection | - | None | None |
|  | SVR12 evaluation | HCV1a |  |  | 174S | None | None |
| Patient 15 | Baseline | HCV1b | No | Relapse | - | 31I/M, 93H | None |
|  | SVR12 evaluation |  |  |  | - | 31M, 93H | None |
| Patient 16 | Baseline | HCV1b | No | Relapse | None | None | None |
|  | SVR12 evaluation |  |  |  | None | None | None |
| Patient 17 | Baseline | HCV1a | Yes | Relapse | None | None | - |
|  | SVR12 evaluation |  |  |  | None | 30R | None |
| Patient 18 | Baseline | HCV1b | No | Reinfection | - | None | None |
|  | SVR12 evaluation |  |  |  | - | 31V, 93H | None |
| Patient 19 | Baseline | HCV3a | Yes | Relapse | - | None | None |
|  | SVR12 evaluation |  |  |  | - | 93H | None |
| Patient 20 | Baseline | HCV1a | No | Relapse | 174S | None | - |
|  | SVR12 evaluation |  |  |  | 168G, 174S | 30R | None |
| Patient 21 | Baseline | HCV4d | Yes | Relapse | None | - | None |
|  | SVR12 evaluation |  |  |  | 168V | - | None |
| Patient 22 | Baseline | HCV1a | Yes | Relapse | 174S | 31M | - |
|  | SVR12 evaluation |  |  |  | 174S | 31M | None |
| Patient 23 | Baseline | HCV1a | Yes | Relapse | 174S | None | - |
|  | SVR12 evaluation |  |  |  | 174S | 31F, 58N | None |
| Patient 24 | Baseline | HCV3a | Yes | Relapse | - | None | None |
|  | SVR12 evaluation |  |  |  | - | None | None |
| Patient 25 | Baseline | HCV1a | Yes | Relapse | 55I, 80K | None | - |
|  | SVR12 evaluation |  |  |  | 55I, 80K | 31M, 58P | None |
| Patient 26 | Baseline | HCV1a | Yes | Relapse | 174S | None | - |
|  | SVR12 evaluation |  |  |  | 174S | None | None |
| Patient 27 | Baseline | HCV1a | No | Relapse | - | None | None |
|  | SVR12 evaluation |  |  |  | 36M, 155K, 174S | 28T | None |
| Patient 28 | Baseline | HCV4a | No | Relapse | - | None | None |
|  | SVR12 evaluation |  |  |  | - | 28V | None |
| Patient 29 | Baseline | HCV3a | Yes | Relapse | - | None | None |
|  | SVR12 evaluation |  |  |  | - | None | None |
| Patient 30 | Baseline | HCV1b | No | Probably relapse | 117H | 93H | - |
|  | SVR12 evaluation |  |  |  | - | 31V, 93H | - |
| Patient 31 | Baseline | HCV1b | No | Relapse | None | 32A, 93H | - |
|  | SVR12 evaluation |  |  |  | None | 93H | - |
| Patient 32 | Baseline | HCV1b | No | Relapse | - | 93H | None |
|  | SVR12 evaluation |  |  |  | None | None | None |
| Patient 33 | Baseline | HCV1a | No | Relapse | 174S | None | - |
|  | SVR12 evaluation |  |  |  | 174G/S | None | None |
| Patient 34 | Baseline | HCV4d | Yes | Relapse | None | None | - |
|  | SVR12 evaluation |  |  |  | None | None | 282T |
| Patient 35 | Baseline | HCV3a | Yes | Relapse | - | None | None |
|  | SVR12 evaluation |  |  |  | - | None | None |
| Patient 36 | Baseline | HCV1a | Yes | Reinfection | 174S | None | None |
|  | SVR12 evaluation | HCV3a |  |  | - | 30K | None |
| Patient 37 | Baseline | HCV3a | Yes | Relapse | - | 30K | None |
|  | SVR12 evaluation |  |  |  | - | 30K | None |
| Patient 38 | Baseline | HCV1a | Yes | Relapse | None | None | - |
|  | SVR12 evaluation |  |  |  | None | None | None |
| Patient 39 | Baseline | HCV4a | Yes | Relapse | - | None | None |
|  | SVR12 evaluation |  |  |  | None | 28M, 30R | None |
| Patient 40 | Baseline | HCV3a | Yes | Relapse | - | None | None |
|  | SVR12 evaluation |  |  |  | - | None | None |
| Patient 41 | Baseline | HCV1a | Yes | Relapse | 80K, 174S | None | - |
|  | SVR12 evaluation |  |  |  | 80K, 168V, 174S | 30R | None |
| Patient 42 | Baseline | HCV4a | Yes | Relapse | None | None | None |
|  | SVR12 evaluation |  |  |  | None | None | - |
| Patient 43 | Baseline | HCV1a | No | Relapse | None | 58D | None |
|  | SVR12 evaluation |  |  |  | None | None | None |
| Patient 44 | Baseline | HCV4a | Yes | Relapse | None | None | None |
|  | SVR12 evaluation |  |  |  | None | None | None |
| Patient 45 | Baseline | HCV3a | No | Relapse | - | None | None |
|  | SVR12 evaluation |  |  |  | - | None | None |
| Patient 46 | Baseline | HCV1a | No | Relapse | - | None | None |
|  | SVR12 evaluation |  |  |  | - | 30E | None |
| Patient 47 | Baseline | HCV3a | No | Relapse | - | None | None |
|  | SVR12 evaluation |  |  |  | - | 93H | None |
| Patient 48 | Baseline | HCV1a | Yes | Relapse | 36L, 54S, 55I, 80L | 30H, 58Y | - |
|  | SVR12 evaluation |  |  |  | 36L, 54S, 55I, 80L | 30H, 58Y | None |
| Patient 49 | Baseline | HCV1b | Yes | Probably relapse | - | None | None |
|  | SVR12 evaluation |  |  |  | - | None | - |
| Patient 50 | Baseline | HCV1a | Yes | Relapse | 170V, 174S | None | None |
|  | SVR12 evaluation |  |  |  | 170V, 174S | None | None |
| Patient 51 | Baseline | HCV1a | Yes | Relapse | 174S | None | None |
|  | SVR12 evaluation |  |  |  | 174S | None | None |
| Patient 52 | Baseline | HCV1a | Yes | Relapse | None | None | None |
|  | SVR12 evaluation |  |  |  | None | None | None |
| Patient 53 | Baseline | HCV1a | Yes | Relapse | - | None | None |
|  | SVR12 evaluation |  |  |  | - | None | None |
